# Supplementary material for: Sex and disease regulate major histocompatibility complex class I expression in human lung epithelial cells
Source: Physiol Rep. 2024 Sep 2;12(17):e70025. doi: 10.14814/phy2.70025 (PMC11368564; doi:10.14814/phy2.70025)
Supplement: Supplementary file 1 — Data S1. [file PHY2-12-e70025-s001.zip › PHYSREP-2024-06-395-T-f08-z-.pdf]

Sex and Disease Regulate Major Histocompatibility Complex class I Expression in Human Lung Epithelial Cells

Mathé Justine, Brochu Sylvie, Adam Damien, Brochiero Emmanuelle and Perreault Claude

Online data supplement

Supplementary Table S1*. Demographics of COPD, IPF, and non-diseased control donors. scRNA-seq data were downloaded from GSEA136831(1). NS: Non-smokers.
*Data are shown in a separate Excel file.

Supplementary Table S2. Sex and age of non-diseased and CF donors included in the scRNA-seq analysis**.**

Donors	Sex	Age
CO	F	18
CO	M	52
CO	?	?
CO	M	47
CO	M	37
CO	M	47
CO	F	63
CO	F	53
CO	M	24
CO	M	52
CO	F	48
CO	?	?
CO	?	?
CO	?	?
CO	?	?
CO	?	?
CO	?	?
CO	?	?
CO	?	?
CF	F	42
CF	F	16
CF	M	30
CF	F	24
CF	F	38
CF	F	23
CF	M	35
CF	F	30
CF	M	37
CF	F	25-30
CF	F	20-25
CF	?	?
CF	F	40-45
CF	F	15-20
CF	F	25-30
CF	M	55-60
CF	M	25-30
CF	F	26-30
CF	?	6

Data were downloaded from GSE150674(2).

Supplementary Table S3*. DEGs between males and females from non-diseased NS, COPD, and IPF donors. scRNA-seq data were downloaded from GSEA136831(1). Sheet 1 shows a non-diseased NS donors comparison, sheet 2 shows a COPD donors comparison, and sheet three shows an IPF donors comparison. NS: Non-smoker; M: Male; F: Female.
*Data are shown in a separate Excel file.

Supplementary Table S4. Demographics of non-diseased (GD) and CF donors analyzed by immunofluorescence staining.

Patient ID	Sex	Age	Smoking status	Infections
GD-71	Female	48	Yes	No
GD-79	Female	66	Yes	No
GD-174	Female	48	No	No
GD-75	Male	24	Yes	No
GD-105	Male	59	No	No
GD-116	Male	48	Yes	No
CF-108	Female	37	No	Yes
CF-112	Female	35	No	Yes
CF-124	Female	45	No	Yes
CF-116	Male	28	No	Yes
CF-117	Male	34	No	Yes
CF-123	Male	34	No	Yes

The Respiratory tissue biobank of the CRCHUM provided cytospins from these donors. Immunofluorescence analysis performed with these samples are shown in Figure 2 and E4.

Supplementary Table S5*. DEGs enriched in GO biological pathways of interest. This table shows the DEGs enriched in the pathways presented in Figure 1 (LECs vs. Lymphoid, myeloid, and fibroblastic cells), Figure 3 (M vs. F ATII and ciliated cells; control donors), and Figure 7 (M vs. F; COPD and IPF donors). M: Male; F: Female.
*Data are shown in a separate Excel file.

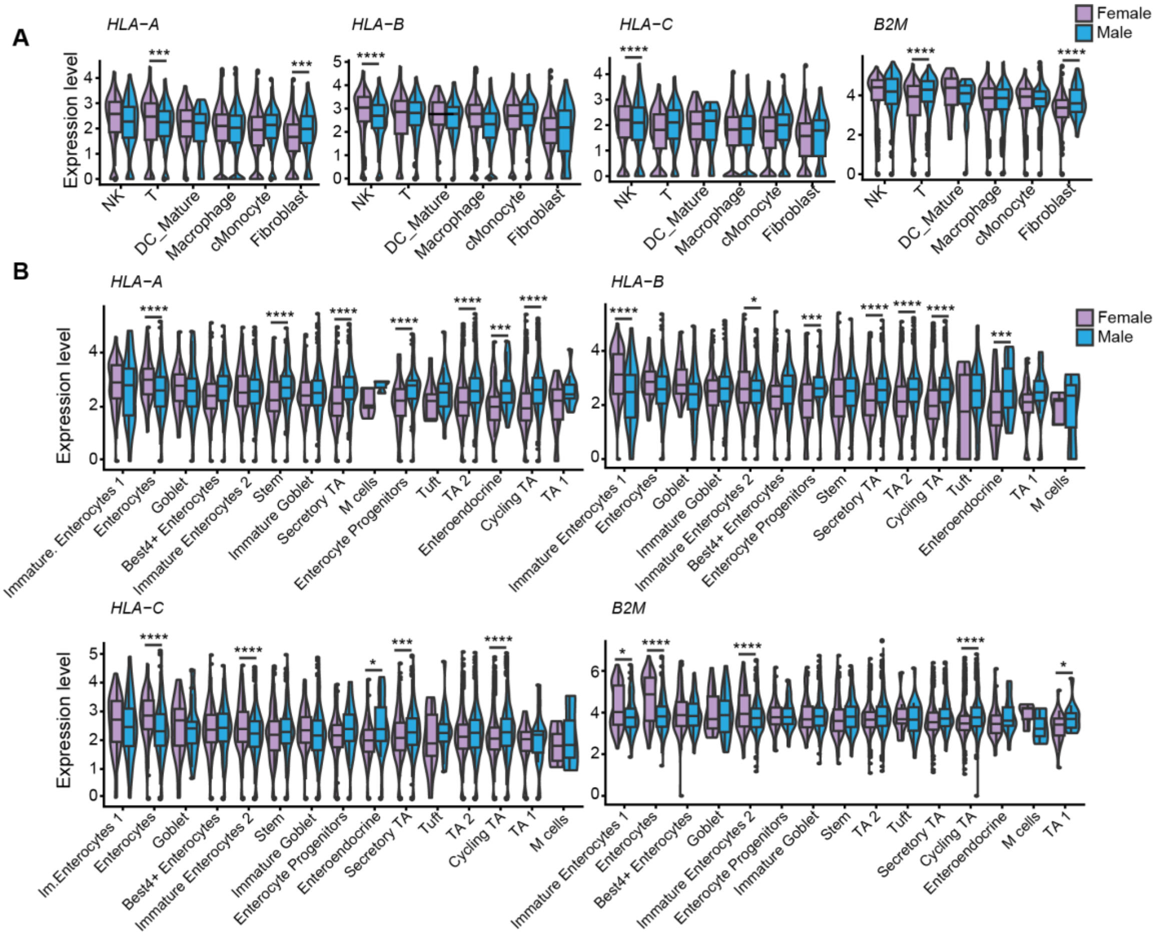

Figure S1. **Sex dimorphism in MHC I expression is found in ECs from the lung and gut. A**) Comparison of MHC I alleles and B2M transcript expression between male (n=10) and female (n=11) non-ECs in 21 non-smoking control donors. **B**) Comparison of MHC I alleles and B2M transcript expression between males and females in intestinal ECs from 12 non-diseased donors (6 males and 6 females). Statistical significance was assessed using a non-parametric Wilcoxon (*$p < 0.05$, **$p < 0.01$, ***$p < 0.001$, ****$p < 0.0001$).

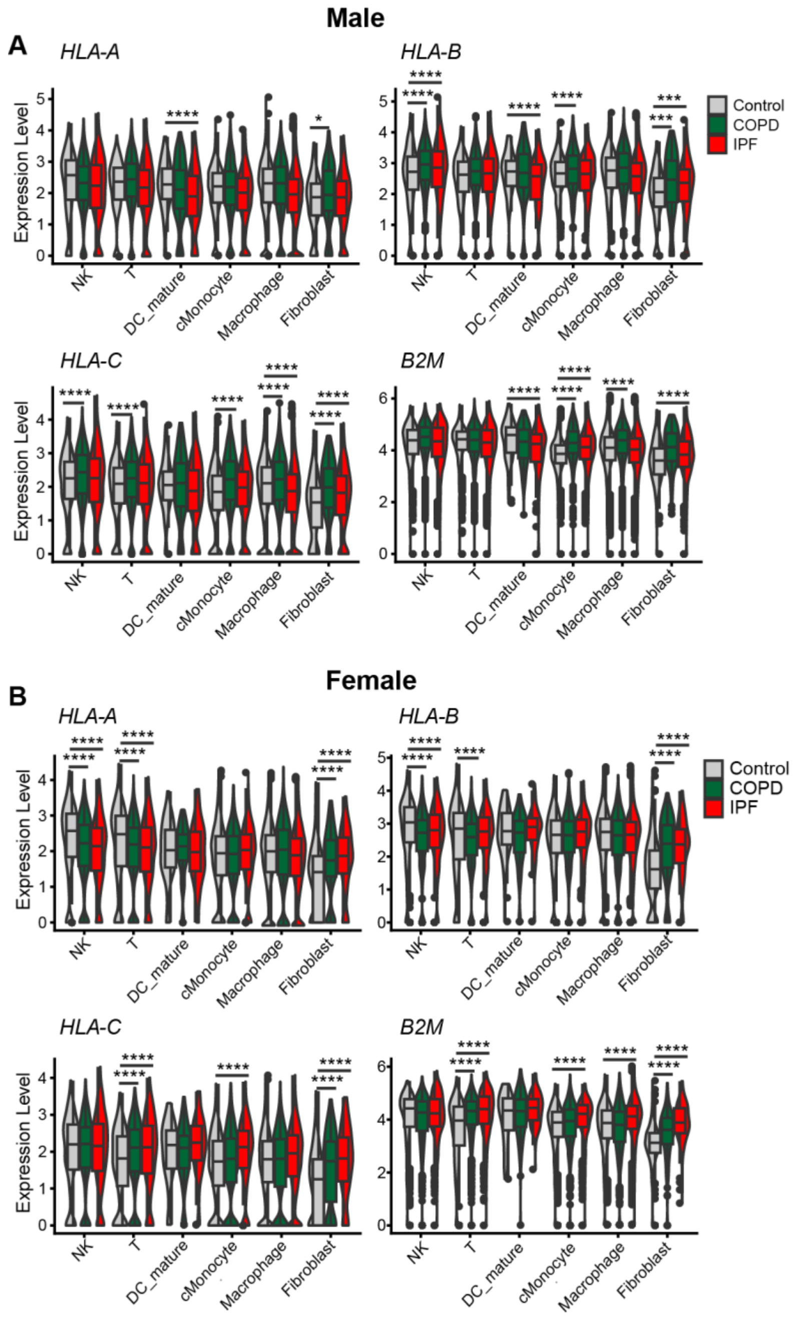

Figure S2. Regulation of MHC I expression in non-epithelial cells from the lung of patients with COPD and IPF. A-B) MHC I expression in non-ECs from male and female lungs with COPD (n= 9 males and 9 females) (green) and IPF (n=22 males and 6 females) (red), compared to control donors (grey). Statistical significance was assessed using a non-parametric Wilcoxon test (**p* < 0.05, ***p* < 0.01, ****p* < 0.001, *****p* < 0.0001). All statistical tests compared control donors with either COPD or IPF donors.

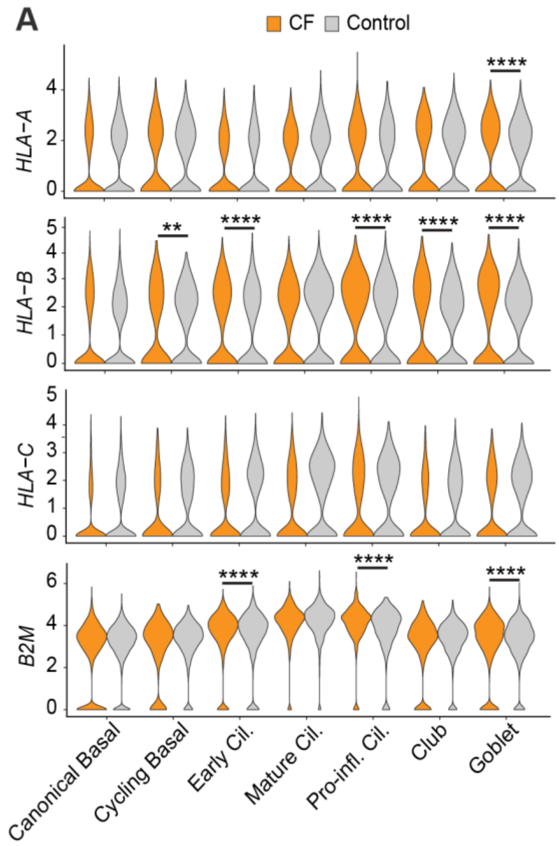

Figure S3. **Secretory and ciliated cells increase MHC I expression in CF donors at the transcriptomic levels. A**) Violin plots show increased MHC I and B2M expression in ECs from CF patients compared to healthy donors (Control). The analysis included 19 Control and 19 CF donors from GSE150674, consisting of male and female donors of various ages (Supplementary Table E2); sex and age information was unavailable for all samples. Statistical significance was determined using a non-parametric Wilcoxon test (*$p < 0.05$, **$p < 0.01$, ***$p < 0.001$, ****$p < 0.0001$).

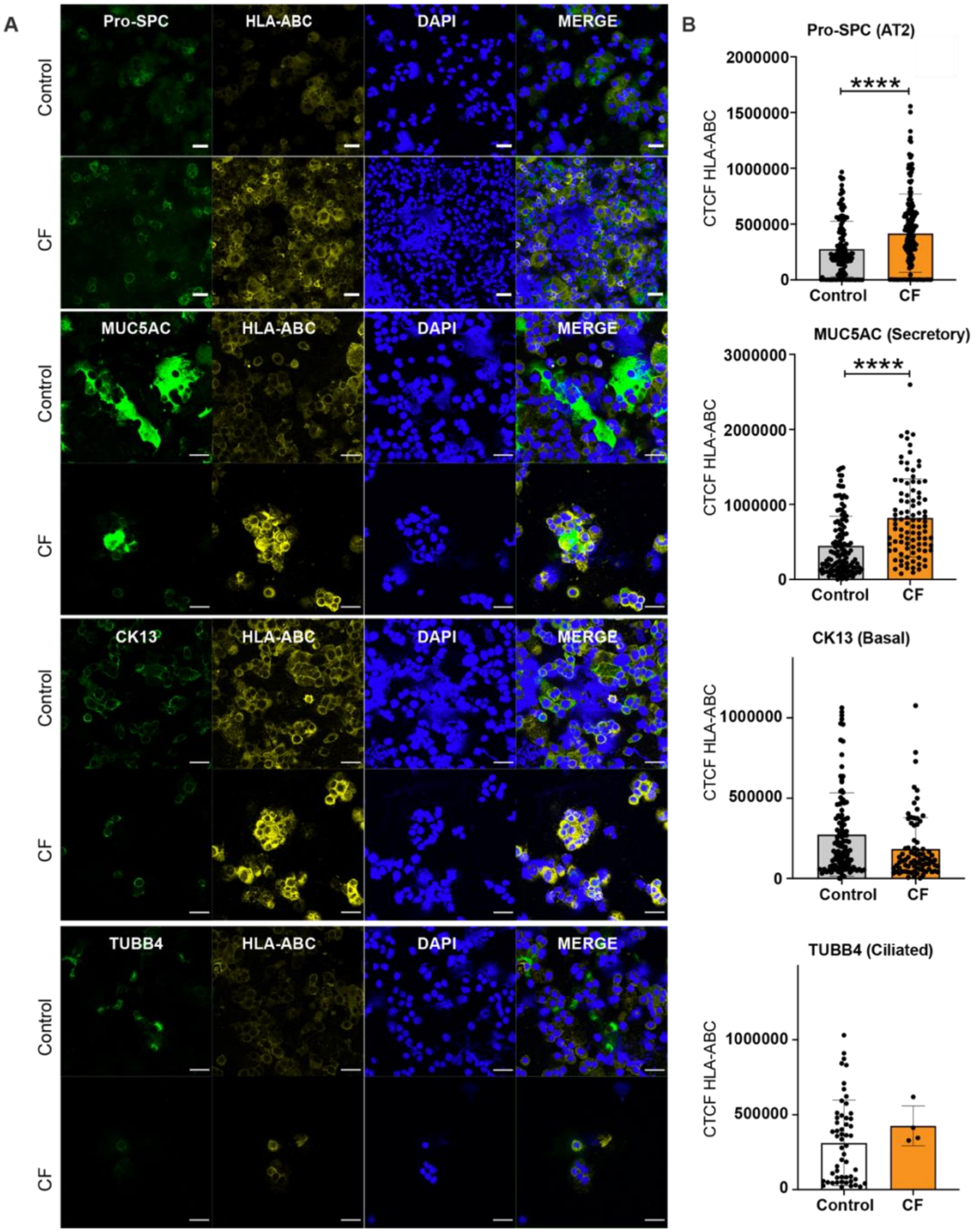

Figure S4. **ATII and secretory cells increase MHC I protein expression in CF donors. A**) Immunofluorescence staining of ATII (Pro-SPC), Secretory (MUC5AC), Basal (CK13), and ciliated (TUBB4) cells with pan anti-HLA-ABC antibody. Representative images for all staining conditions. Images were taken using a 40X objective. Scale bars, 30μm. All donors (CF and control) were stained and acquired simultaneously in the same conditions for each marker. No changes in the intensity or contrast were made. **B**) CTCF value, as defined in Figure 2B. Statistical significance was assessed using an unpaired T-test (*p <

0.05, **$p < 0.01$, ***$p < 0.001$, ****$p < 0.0001$). Graphs show the means of all cells for each group ± SD. 6 donors were analyzed per condition (Control and CF).

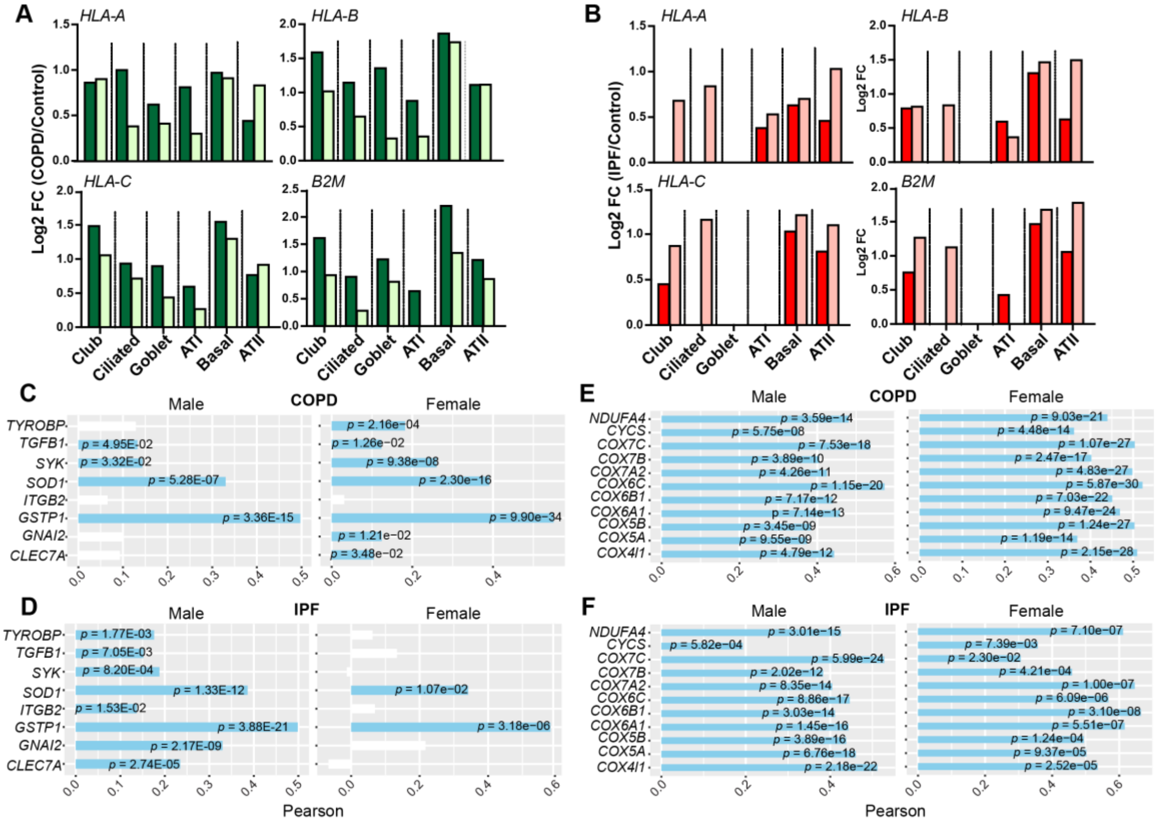

Figure S5. **Differential regulation of MHC I and ROS-related transcripts in males and females with COPD and IPF. A-B**) Log2 fold-change (FC) of MHC I expression in COPD (n= 9 males and 9 females) (A) or IPF (n=22 males and 6 females) (B) donors compared to their healthy counterparts in both males (darker panel) and females (lighter panel). **C-D**) Pearson correlation of genes included in the "positive regulation of superoxide anion generation" pathway with HLA-B expression in ATII with COPD (C) and IPF (D). **E-F**) Pearson correlation of genes included in the "mitochondrial electron transport, cytochrome c to oxygen" pathway with HLA-B expression in ATII with COPD (E) and IPF (F). All left panels show male data and the right panels show female data.

References

1. Adams TS, Schupp JC, Poli S, Ayaub EA, Neumark N, Ahangari F, *et al.* Single-cell RNA-seq reveals ectopic and aberrant lung-resident cell populations in idiopathic pulmonary fibrosis. *Science Advances* 2020;6:eaba1983.
2. Carraro G, Langerman J, Sabri S, Lorenzana Z, Purkayastha A, Zhang G, *et al.* Transcriptional analysis of cystic fibrosis airways at single-cell resolution reveals altered epithelial cell states and composition. *Nat Med* 2021;27:806–814.
