# Supplementary material for: Sex and disease regulate major histocompatibility complex class I expression in human lung epithelial cells
Source: Physiol Rep. 2024 Sep 2;12(17):e70025. doi: 10.14814/phy2.70025 (PMC11368564; doi:10.14814/phy2.70025)
Supplement: Supplementary file 1 — Data S1. [file PHY2-12-e70025-s001.zip › PHYSREP-2024-06-395-T-file003.docx]

**Supplementary Table S2. Sex and age of non-diseased and CF donors included in the scRNA-seq analysis.**

| **Donors** | **Sex** | **Age** |
| --- | --- | --- |
| **CO** | **F** | **18** |
| **CO** | **M** | **52** |
| **CO** | **?** | **?** |
| **CO** | **M** | **47** |
| **CO** | **M** | **37** |
| **CO** | **M** | **47** |
| **CO** | **F** | **63** |
| **CO** | **F** | **53** |
| **CO** | **M** | **24** |
| **CO** | **M** | **52** |
| **CO** | **F** | **48** |
| **CO** | **?** | **?** |
| **CO** | **?** | **?** |
| **CO** | **?** | **?** |
| **CO** | **?** | **?** |
| **CO** | **?** | **?** |
| **CO** | **?** | **?** |
| **CO** | **?** | **?** |
| **CO** | **?** | **?** |
| **CF** | **F** | **42** |
| **CF** | **F** | **16** |
| **CF** | **M** | **30** |
| **CF** | **F** | **24** |
| **CF** | **F** | **38** |
| **CF** | **F** | **23** |
| **CF** | **M** | **35** |
| **CF** | **F** | **30** |
| **CF** | **M** | **37** |
| **CF** | **F** | **25-30** |
| **CF** | **F** | **20-25** |
| **CF** | **?** | **?** |
| **CF** | **F** | **40-45** |
| **CF** | **F** | **15-20** |
| **CF** | **F** | **25-30** |
| **CF** | **M** | **55-60** |
| **CF** | **M** | **25-30** |
| **CF** | **F** | **26-30** |
| **CF** | **?** | **6** |

Data were downloaded **from** GSE150674(2).
