# Supplementary material for: Sex and disease regulate major histocompatibility complex class I expression in human lung epithelial cells
Source: Physiol Rep. 2024 Sep 2;12(17):e70025. doi: 10.14814/phy2.70025 (PMC11368564; doi:10.14814/phy2.70025)
Supplement: Supplementary file 1 — Data S1. [file PHY2-12-e70025-s001.zip › PHYSREP-2024-06-395-T-file005.docx]

**Supplementary Table S4. Demographics of non-diseased (GD) and CF donors analyzed by immunofluorescence staining.**

| **Patient ID** | **Sex** | **Age** | **Smoking status** | **Infections** |
| --- | --- | --- | --- | --- |
| **GD-71** | **Female** | **48** | **Yes** | **No** |
| **GD-79** | **Female** | **66** | **Yes** | **No** |
| **GD-174** | **Female** | **48** | **No** | **No** |
| **GD-75** | **Male** | **24** | **Yes** | **No** |
| **GD-105** | **Male** | **59** | **No** | **No** |
| **GD-116** | **Male** | **48** | **Yes** | **No** |
| **CF-108** | **Female** | **37** | **No** | **Yes** |
| **CF-112** | **Female** | **35** | **No** | **Yes** |
| **CF-124** | **Female** | **45** | **No** | **Yes** |
| **CF-116** | **Male** | **28** | **No** | **Yes** |
| **CF-117** | **Male** | **34** | **No** | **Yes** |
| **CF-123** | **Male** | **34** | **No** | **Yes** |

**The Respiratory tissue biobank of the CRCHUM provided cytospins from these donors. Immunofluorescence analysis performed with these samples are shown in Figure 2 and E4.**
